# Supplementary material for: A Fatal Case of Native Valve Endocarditis with Multiple Embolic Phenomena and Invasive Methicillin-Resistant Staphylococcus aureus Bacteremia: A Case Report from the Maldives
Source: Trop Med Infect Dis. 2023 Jan 10;8(1):53. doi: 10.3390/tropicalmed8010053 (PMC9865154; doi:10.3390/tropicalmed8010053)
Supplement: Supplementary file 1 [file tropicalmed-08-00053-s001.zip › tropicalmed-2112471-supplementary.pdf]

Tropical Medicine and Infectious Disease

Case Report

A fatal case of native valve endocarditis with multiple embolic phenomena and invasive methicillin-resistant *Staphylococcus aureus* bacteremia: a case report from the Maldives.

Ali Shafeeq[1], Hisham Ahmed Imad[2,3,4]*, Ahmed Azhad[1], Migdhaadh Shareef[1], Mohamed Shaneez Najmy[1], Mohamed Mausool Siraj[1], Mohamed Sunil[1], Rimsha Rafeeu[5], Aishath Sofa Moosa[5], Ahmed Shaheed[6], Thundon Ngamprasertchai[3,7], Wasin Matsee[3,7], Pyae Linn Aung[2], Wang Nguitragool[2,8] and Tatsuo Shioda[4]

[1] National Cardiac Center, Indira Gandhi Memorial Hospital, Malé, Maldives
[2] Mahidol Vivax Research Unit, Faculty of Tropical Medicine, Mahidol University, Bangkok 10400, Thailand
[3] Thai Travel Clinic, Hospital for Tropical Diseases, Bangkok 10400, Thailand
[4] Center for Infectious Disease Education and Research, Department of Viral Infections, Research Institute for Microbial Diseases, Osaka University, Suita 565-0871, Osaka, Japan
[5] Trauma and Emergency, Indhira Gandhi Memorial Hospital, Malé 2002, Maldives
[6] Department of Medicine, Indhira Gandhi Memorial Hospital, Malé 2002, Maldives
[7] Department of Clinical Tropical Medicine, Faculty of Tropical Medicine, Mahidol University, Bangkok 10400, Thailand
[8] Department of Molecular Tropical Medicine and Genetics, Faculty of Tropical Medicine, Mahidol University, Bangkok 10400, Thailand

* Correspondence: hishamahmed.ima@mahidolac.th or imad@biken.osaka-u.ac.jp

Table S1 Antibiotic sensitivity pattern of *S. aureus* isolated from blood culture after 72 hours of incubation.

Organism	Antibiotic Class	Antibiotics	MIC (μg/mL)	Interpretation
Staphylococcus aureus	Beta-lactam	Oxacillin	≥ 4.0	Resistant
Staphylococcus aureus	Beta-lactam	Benzylpenicillin	≥ 0.5	Resistant
Staphylococcus aureus	Aminoglycoside	Gentamicin	≤ 0.5	Sensitive
Staphylococcus aureus	Quinolone	Ciprofloxacin	≤ 0.5	Sensitive
Staphylococcus aureus	Fluoroquinolone	Levofloxacin	0.25	Sensitive
Staphylococcus aureus	Glycopeptide	Vancomycin	1.0	Sensitive
Staphylococcus aureus	Oxazolidinones	Linezolid	2.0	Sensitive
Staphylococcus aureus	Antimycobacterials	Rifampicin	≤ 0.03	Sensitive
Staphylococcus aureus	Glycycline	Tigecycline	≤ 0.12	Sensitive
Staphylococcus aureus	Lipopeptide	Daptomycin	≤ 1.0	Sensitive
Staphylococcus aureus	Tetracycline	Tetracyclines	≤ 1.0	Sensitive
Staphylococcus aureus	Lincosamide	Clindamycin	≤ 0.5	Sensitive

MIC: Minimal inhibitory concentration

Table S2. Modified Dukes criteria for infective endocarditis

**Major criteria**	**Case presented**
Supportive laboratory evidence[a]	Positive blood cultures for MRSA

Evidence of endocardial involvement[b]	Vegetation on mitral valve
**Minor criteria**	
Predisposing heart condition or IVDU	Active IVDU
Fever > 38°C	Febrile at presentation
Vascular phenomenon[c]	Multiple Janeway lesions present
Immunological phenomenon[d]	Osler's nodes and Roths spots present
Positive blood culture not meeting the Major criterion[e]	-

[a] Typical microorganism of infective endocarditis from two separate blood cultures: Viridians streptococci, Staphylococcus aureus, Streptococcus bovis, HACEK group or community acquired enterococci, in the absence of a primary focus. Additional a single positive culture for Coxiella brunetii or serology (antibody titire >1:800

[b] Echocardiogram supportive of infective endocarditis. Definition of positive findings: Oscillating intracardiac mass, on valve or supporting structures, or in the path of regurgitation jets, or on implanted material, in the absence of an alternative anatomic explanation or myocardial abscess or new partial dehiscence of prosthetic valves. New valvular regurgitation.

[c] Major arterial emboli, septic pulmonary infarcts, mycotic aneurysm, intracranial hemorrhage, conjunctival hemorrhage, Janeway lesions.

[d] Glomerulonephritis, Osler's nodes, Roths spots, rheumatoid factor

[e] Excluding single positive cultures for coagulase-negative staphylococci and organism that do not cause endocarditis or serological evidence of active infection with organism consistent with infective endocarditis

IVDU: intravenous drug use, MRSA: methicillin-resistant *Staphylococcus aureus*

This table was obtained from a text book. "Infective endocarditis: a multidisciplinary approach. ISBN: 978-0-12-820657-7

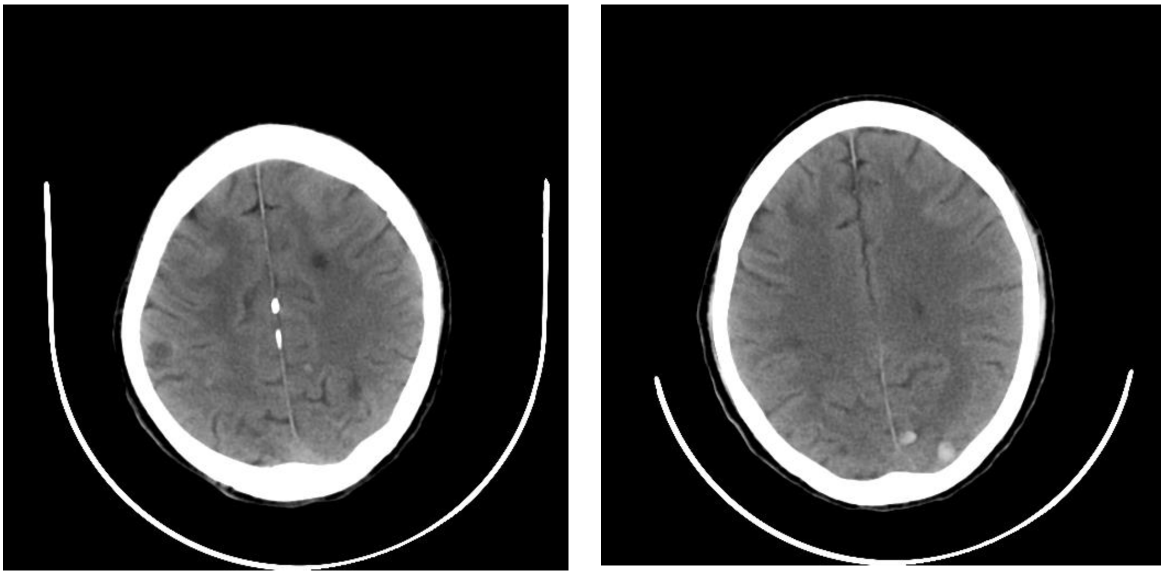

(**a**) (**b**)

Figure S1. Computed tomography images depicting the coronal view of the brain (**a)** Bilateral multiple subcortical hypodense foci representing infarctions (**b**) Unilateral hyperdense foci consistent with hemorrhagic transformation.

Table S3. Comparison of the case presented with other similar cases of native valve infective endocarditis amongst intravenous drug user.

	Present case	Other cases	
		Case-1[35]	Case-2[36]
General characteristics	• 31-year-old male from the Maldives<br>• IVDU (+)<br>• No prior history of IE	• 26-year-old male from USA<br>• IVDU (+)<br>• No prior history of IE	• 36-year-old female from USA<br>• IVDU (+)<br>• No prior history of IE
Presenting complaints	• Referred as a case of septicemia (Adjunct with a four days' history of fever fol-	• Fever and cough for a week	• Fever and cough for two weeks

	lowed by decreased conscious level)		
Gasglow Coma Scale	• 10/15	15/15	Not provided
Vitals at presentation	• Temperature: 38.1° C<br>• Pulse: 142 beats per min.<br>• Blood pressure: 88/58 mmHg<br>• Respiratory rate: 52 breaths per min.	• Hemodynamically stable	• Pulse: 124 beats per min.<br>• Blood pressure: 112/59 mmHg<br>• Respiratory rate: 27 breaths per min.
Stigmata of infective endocarditis	Janeway lesion<br>Osler's nodes<br>Splinter hemorrhages	Janeway lesion<br>Osler's nodes	Not present
Cardiovascular examinations findings	• Tachycardia<br>• Pansystolic murmur (Grade-3 intensity)	• Regular rate and sinus rhythm<br>• No murmur	• Holo systolic murmur
Echocardiographic findings	• Vegetation (30 x 18mm) on mitral valve<br>• Ruptured chordae tendinae Mitral regurgitation	• Vegetation (15 x 13mm) on tricuspid valve	• Vegetation (18 x 24mm) on tricuspid valve
Hematological and inflammatory marker	• Leukocytosis with thrombocytopenia<br>• Elevated CRP	• Not provided	• Leukocytosis
Isolated micro-organism from blood	MRSA	MRSA	MRSA
Other complications	• Septic embolization to brain, eye and extremities	• Septic embolization to lungs, spleen, kidneys, prostate, bladder, and extremities	• Septic embolization to lungs
Case mangement	• Inotropic support<br>• Mechanical ventilation<br>• Vancomycin 20mcg/mL	• Excision of mitral valve left leaflet vegetation's and repair of mitral valve perforation.<br>• Excision of the tricuspid valve chordal vegetation and repair of tricuspid valve chordal rupture<br>• Repair of mitral valve perforation<br>• Vancomycin, piperacillin-tazobactam, daptomy-	• Vancomycin, piperacillin-tazobactam, clindamycin<br>• Percutaneous extraction of the tricuspid valve vegetation using suction filtration and veno-venous bypass<br>• Aspiration of the right knee

		cin	
Outcome	• Demise	• Discharge after six weeks	• Discharge after six weeks and lost to follow-up
